# Not Able to Lead a Healthy Life When You Need It the Most: Dual Role of Lifestyle Behaviors in the Association of Blurred Work-Life Boundaries With Well-Being

**DOI:** 10.3389/fpsyg.2020.607294

**Published:** 2020-12-23

**Authors:** Helen Pluut, Jaap Wonders

**Affiliations:** ^1^Department of Business Studies, Leiden University, Leiden, Netherlands; ^2^SMC Rijnland Fysiotherapeuten, Leiden, Netherlands

**Keywords:** blurred work-life boundaries, emotional exhaustion, lifestyle, happiness, well-being, COVID-19

## Abstract

As there is a growing trend for people to work from home, precipitated by the COVID-19 pandemic, this research examines the impact of blurred work-life boundaries on lifestyle and subjective well-being. Our cross-sectional study in the Netherlands demonstrates that heightened levels of blurred work-life boundaries predict negative changes in happiness through enhanced emotional exhaustion. In addition, the findings point to a dual role of lifestyle in this process. On the one hand, we observed that healthy overall lifestyle patterns buffered employees against the detrimental effects of blurred work-life boundaries and emotional exhaustion on happiness. On the other hand, employees who experienced increases in blurring of work-life boundaries reported a deterioration in healthy lifestyle behaviors, which in turn was related to reduced happiness. Paradoxically, it seems that those who would benefit the most from a healthy lifestyle are less able to sustain health-promoting behaviors. A case for shared responsibility between employers and employees is built as we discuss the practical implications of the current research.

## Introduction

When COVID-19 became a pandemic and an increasingly high number of people got infected also in the Netherlands, the Dutch government announced an intelligent lockdown. As of March 15, 2020, Dutch citizens were advised to keep 1.5 m distance from each other, gatherings with more than three persons other than household members were prohibited, and employees were urged to work from home as much as possible (Rijksoverheid, 2020). Lockdown measures worldwide have inherently created a large-scale working from home experiment, which is considered by many a big success (Kelly, 2020). In light of positive noises about working from home and expectations that working from home might become the new norm in the wake or continuation of the COVID-19 pandemic (Kamouri and Lister, 2020; Lopez-Leon et al., 2020), it is imperative to examine its implications for lifestyle and employee well-being.

Dutch observational studies have shown – and newspapers reported – that the COVID-19 lockdown has a non-negligible impact on people’s lifestyle in terms of fewer social contacts, changed eating habits, decreased physical activity, decreased sleep quality, and psychological factors (iResearch, 2020). Cross-sectional studies in countries like Australia (Stanton et al., 2020), China (He et al., 2020), Chile (Reyes-Olavarría et al., 2020), Italy (Di Renzo et al., 2020), and France (Constant et al., 2020) confirm that, during the COVID-19 lockdown, people have gained weight, engaged less in physical activity, showed more sedentary behavior, consumed more alcohol, slept less well, and changed their eating habits (both positive and negative changes in healthy nutrition have been observed). A study conducted in Spain showed that changes in health-related behaviors depend on the duration of confinement due to COVID-19 and people may gradually improve their lifestyle during confinement (López-Bueno et al., 2020). We set out to contribute to this recent stream of research on changes in lifestyle since the onset of COVID-19. Our cross-sectional study focuses on working people in the Netherlands and aims to elucidate the associations between work, happiness, and lifestyle during the COVID-19 lockdown. Ultimately, our goal is to better understand the role of lifestyle in employee well-being.

Notwithstanding the obvious benefits of working from home (e.g., flexibility, no commute), it may also pose a risk for employees’ health and subjective well-being. Previous COVID-19-related research argues that working from home is related to psychosocial risks such as exhaustion and boredom (Brooks et al., 2020) as well as social isolation in the professional sphere and blurring of boundaries (Bouziri et al., 2020). Following recent calls by Cho (2020) and O’Connor et al. (2020) for more research on the impact of remote and flexible working arrangements and on changes in role boundaries in the context of COVID-19, we focus in this study on the blurring of work-life boundaries and its consequences for employee happiness. As many are forced to work from home on a permanent basis during the COVID-19 lockdown, it may become difficult to tell when the workday begins and ends. Employees may be tempted to engage in work-family multitasking more than usual as they attend to personal or family matters during the workday. In essence, the work-from-home practice in the age of COVID-19 is associated with a fungible use of time (Borpujari et al., 2020) and evidently creates considerable challenges for work-life balance (Cho, 2020). Another expected consequence of the ongoing COVID-19 outbreak is that it “may modify, for better or for worse, lifestyle behaviors” (Balanzá-Martínez et al., 2020, p. 399). In this paper, we aim to advance our understanding of the role of lifestyle in how employees respond to blurred work-life boundaries. To this end, we propose and test a conceptual model that integrates mediating and moderating perspectives on lifestyle in the process by which blurred boundaries can reduce employee happiness.

## Theory and Hypotheses

Well-being is a multifactorial construct and can be understood broadly as a person’s eudaimonic experience of fulfillment and purpose or the hedonic experience of feeling good (Linley et al., 2009; Di Fabio and Palazzeschi, 2015). Psychological well-being, which is the eudaimonic approach to well-being, is seen as well-being related to the full functioning of a person (Ryff and Keyes, 1995). The hedonic approach focuses on subjective well-being, which is defined as the balance between positive and negative mood and the evaluation of a person’s satisfaction with life (Linley et al., 2009). In colloquial terms, subjective well-being is often referred to as happiness (Diener et al., 2018; Myers and Diener, 2018). In this study, we consider happiness an important outcome in and of itself because it is a subjective state of mind reflecting overall subjective well-being (Diener, 2000) as well as a key indicator of career sustainability among employees (De Vos et al., 2020).

We draw on border theory (Clark, 2000) to argue that a particularly likely consequence of working from home as the new norm amid the COVID-19 pandemic is blurring of boundaries between work and private life (see also Schieman and Young, 2010; Bouziri et al., 2020; Cho, 2020). With greater role integration and blurring of boundaries, family-to-work and work-to-family conflict are more likely as well as interruptions and distractions while working and role transitions (Desrochers et al., 2005). When work (persistently) encroaches on and intrudes personal or family time, it might become more difficult to psychologically detach from work. Moreover, stress and negative emotions may arise from the struggles to attain or maintain work-life balance. Central to border theory is the notion that individuals are motivated to create a desired balance between work and other domains (Clark, 2000). In accordance with this notion, it has been found that the work-home interface influences how happy and satisfied individuals are with their lives (Odle-Dusseau et al., 2012; Sirgy and Lee, 2018; Tang et al., 2020). We therefore propose that blurred work-life boundaries reduce employee happiness.

To illuminate the pathways through which blurred work-life boundaries result in reduced happiness, we focus on emotional exhaustion as a mediating mechanism. Emotional exhaustion is a component of burnout and is characterized by a negative state of physical and emotional depletion due to work (Maslach and Jackson, 1981). We propose that border creep in the work-home interface is an emotionally exhausting experience that may reduce employee happiness. Indeed, those who experience difficulties with their work-life balance are more emotionally exhausted (Umene-Nakano et al., 2013). To the best of our knowledge, emotional exhaustion has not been examined as a consequence of blurred work-life boundaries *per se*. However, blurring of boundaries is positively associated with work-family conflict (Desrochers et al., 2005; Glavin and Schieman, 2012) and work-family conflict has been extensively linked to emotional exhaustion (for a review, see Allen et al., 2000). Moreover, emotional exhaustion has negative effects on happiness (Eckleberry-Hunt et al., 2016; Peralta and Saldanha, 2017). Thus, we put forward the following mediation hypothesis:

Hypothesis 1: Blurring of work-life boundaries is negatively associated with employee happiness through increased emotional exhaustion.

In this paper, lifestyle is conceptualized as comprising four behaviors: sleep, nutrition, physical activity, and relaxation (as a psychosocial behavior to cope with stress). Studies on lifestyle have extensively focused on (a combination of) sleep (e.g., Agarwal et al., 2020; Ingram et al., 2020), physical activity (Griep et al., 2015; Larsson et al., 2019), nutrition (Wu et al., 2016; Nishi et al., 2017), and psychosocial behaviors such as relaxation (e.g., Strijk et al., 2009; Van Scheppingen et al., 2015). These four factors are also included in assessment toolkits (Reis et al., 2019) and guidelines for lifestyle (Kanji et al., 2018).

A substantial body of research demonstrates the impact of lifestyle on mental health and subjective well-being (Walsh, 2011; Prendergast et al., 2016; Atzendorf et al., 2018; Lianov et al., 2019; Wang and Geng, 2019; Ingram et al., 2020). Specifically, a healthier diet (Piqueras et al., 2011; Blanchflower et al., 2013; Rooney et al., 2013; White et al., 2013), higher quantity and quality of sleep (Shin and Kim, 2018; Zhao et al., 2019) and more physical activity (Khazaee-Pool et al., 2015; Murphy et al., 2018; Zhang and Chen, 2019) have been shown to lead to higher happiness. Thus, research has established that healthy lifestyle behaviors can make people happier.

**FIGURE 1:**
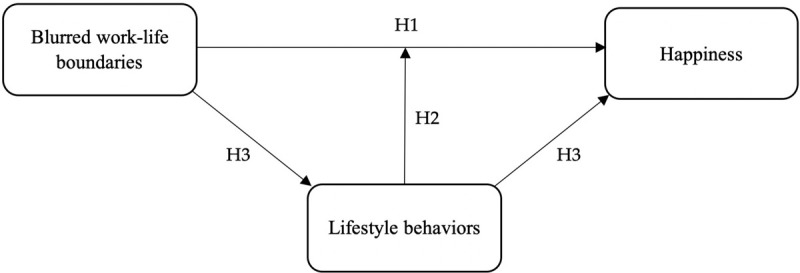
Conceptual model and overview of hypotheses.

However, the role of lifestyle in the process by which blurred work-life boundaries influence happiness remains elusive. Drawing a parallel to the stressor-detachment model (Sonnentag and Fritz, 2015), lifestyle can be considered both a mediator and a moderator in this process. The mediation model proposes that blurred boundaries (perhaps via emotional exhaustion) impair healthy lifestyle behaviors, and in turn, lifestyle risk factors negatively influence happiness. It would imply that an unhealthy lifestyle is an explanation for why blurred work-life boundaries (and emotional exhaustion) reduce happiness. The moderation model proposes that healthy lifestyle behaviors buffer the (indirect) effect of blurred work-life boundaries on happiness. The relative merits of these models are not trivial; whereas a buffering effect implies that a healthy lifestyle has the potential to prevent blurred boundaries from lowering happiness, the mediation model suggests that a most likely consequence of blurred boundaries is a less healthy lifestyle. Our conceptual model in Figure 1 integrates both perspectives.

Empirical results on the potential buffering effects of lifestyle behaviors are scant and sometimes inconsistent (cf. Gerber and Pühse, 2008; DeBoer et al., 2012). However, lifestyle has been related to functional coping (Erschens et al., 2018) and recovery (Rook and Zijlstra, 2006) in the context of work-related stress. When blurred boundaries and emotional exhaustion result in a loss of resources, additional or substitute resources can be gained through a healthy lifestyle (e.g., high sleep quality; Gombert et al., 2018). That is, healthy lifestyle behaviors can help tackle feelings of frustration and fatigue related to work-life issues such that employees are less negatively affected in terms of lower happiness. Insights from the field of psychoneuroimmunology suggest that health-promoting lifestyle behaviors can help individuals increase their psychological resilience by improving their psychoneuroimmunological functions (Kim and Su, 2020). More specifically, exercise and relaxation protect against psychological distress by decreasing the release of stress-related hormones (Moraes et al., 2018), healthy nutrition decreases distress via the gut-brain axis (Madison and Kiecolt-Glaser, 2019), and adequate sleep reduces distress through changes in inflammation-related genes (Chiang et al., 2019). In sum, lifestyle behaviors can be considered adaptive coping strategies that reduce reactivity to distress and loss of resources. We therefore hypothesize that the strength of the blurred boundaries—emotional exhaustion—happiness association varies as a function of lifestyle.

Hypothesis 2: Healthy lifestyle behaviors moderate the indirect association between blurred work-life boundaries and employee happiness via emotional exhaustion such that this indirect association is less strong for employees with a healthier lifestyle than for those with a less healthy lifestyle.

Only a few studies consider lifestyle behaviors as mediators between work factors and well-being (e.g., Allen and Armstrong, 2006). Work stress and job strain have been found to be associated with fewer health-promoting lifestyle behaviors (Lindquist and Cooper, 1999; Lee et al., 2011; Griep et al., 2015; Miron and Colosi, 2018). In response to stress, employees may be motivated to use unhealthy behaviors to manage the aversive state (Ng and Jeffery, 2003). Similarly, it stands to reason that struggles in the work-life sphere and emotional exhaustion will disincline individuals to engage in health-promoting lifestyle behaviors. In fact, it has been shown that stress related to the work-life interface is associated with a variety of unhealthy lifestyle behaviors (for a review, see Hammer and Sauter, 2013). Moreover, Wu et al. (2016) found that those with a poor work-recreation balance (as part of work-life balance) are more likely to exhibit an unhealthy lifestyle, including poor nutrition, less sports and exercise activities, and poor stress management. Emotional exhaustion has also been linked to unhealthy lifestyle choices (Petrelli et al., 2018; Padilla et al., 2019). Conceptually, blurred boundaries and emotional exhaustion may deplete resources (i.e., self-regulatory resources but also time) that could facilitate a health-promoting lifestyle. Although unhealthy behaviors such as indulgent eating can bring instant gratification, as a form of maladaptive coping (Lindquist and Cooper, 1999) it may negatively influence happiness on the longer term. We therefore put forward the following hypothesis:

Hypothesis 3: (a) Blurring of work-life boundaries and (b) emotional exhaustion are negatively associated with employee happiness through less healthy lifestyle behaviors.

## Materials and Methods

### Participants and Procedures

We recruited adults working in the Netherlands through our personal and professional networks, using a convenience sampling strategy. Participants were approached through e-mail letters and posts on social media platforms like LinkedIn soliciting participation in the survey study. The invitation indicated the scope and purpose of the study. We required that individuals were 18 years or older and had been working in the Netherlands over the past few months in order to qualify for participation in this study. Taking the survey was completely voluntary. Before the start of the survey, participants were informed that their data would be used in scientific research and we ensured that all data would be treated anonymously and confidentially. No supplementary advice or approval from the local ethics committee was required. Data were collected during the months of June and July in 2020 (first wave of COVID-19) via online surveys hosted by Qualtrics. A total of 972 individuals participated in our study. We excluded 95 participants who did not meet the eligibility criteria (e.g., unemployed or not residing in the Netherlands) or who provided only demographic information, resulting in a sample size of 877 participants. Effective sample sizes for analyses are somewhat lower and vary based on the number of valid observations for the various study variables (see notes to the tables). Sixty-four percent of the participants were women. The average age of the participants was 41.7 years (*SD* = 12.0). The majority of participants was cohabitating (78.4%) and married or in a relationship (81.4%). Forty-three percent had at least one child living at home. Within this group of families, most had two children and the average age of the youngest child was 9.1 years (*SD* = 6.9). Some participants (12.8%) had the primary responsibility for caring for parents or other relatives. The vast majority of participants (84.4%) had completed higher vocational education or university education. On average, they had been with their current organization for 3.4 years (*SD* = 1.5). The majority of participants were wage-earning workers, either on a permanent contract (60.9%) or temporary contract (22.6%), while the remainder of participants was self-employed. Participants held jobs in a variety of sectors, such as academia, healthcare and ICT, and these jobs ranged from secretarial to managerial.

### Measures

Our survey assessed a variety of variables related to work, lifestyle, and well-being. All survey items were translated to Dutch using the method of back-translation (Brislin, 1970). Respondents could opt for the Dutch or English version in line with their language preferences. Respondents were instructed to compare the periods before and during the corona crisis, which involves a retrospective analysis. It was communicated to respondents that 15 March 2020 was considered the start of the corona crisis due to the (intelligent lockdown) measures announced on that date in the Netherlands (Rijksoverheid, 2020). Respondents were asked to evaluate statements as they applied to them during the corona crisis, using a Likert scale ranging from 1 = *much less than usual* through 3 = *same as usual* to 5 = *much more than usual.* This response scale has been used previously in for instance the widely used General Health Questionnaire (Goldberg, 1972) and fits our purpose of assessing – with a few exceptions – changes in work, lifestyle, and well-being as a consequence of the COVID-19 lockdown. In line with the conceptual model and hypotheses, our main study variables are blurring of work-life boundaries, emotional exhaustion, happiness, and lifestyle (behaviors).

#### Blurring of Work-Life Boundaries

To measure blurring of boundaries between work and private life, we used three items from Kossek and Lautsch’ (2008) self-assessment tool on flexstyles that showed clear resemblance with the Work-Family Integration-Blurring Scale by Desrochers et al. (2005). We applied a broad focus on family and personal life and ensured that all items were worded in terms of blurring or integration rather than separation. Our items read as follows: “It is difficult to tell where my work life ends and my personal or family life begins,” “I attend to personal or family issues during the workday,” and “During my workday, there is blurring of boundaries between time spent on work and time spent on personal activities.” Cronbach’s alpha for this scale was 0.72.

#### Emotional Exhaustion

We measured emotional exhaustion with three items from the Maslach Burnout Inventory (Maslach and Jackson, 1981) that fit the setting of the current study, namely “I feel emotionally drained from my work,” “I feel fatigued when I get up in the morning and have to face another day on the job,” and “I feel frustrated by my job.” The internal consistency for this scale was 0.82.

#### Happiness

Happiness can be used as a practicable and subjective measure to identify hedonic well-being (Di Fabio and Palazzeschi, 2015; Diener et al., 2018; Trudel-Fitzgerald et al., 2019). We evaluated happiness using a single item from the General Health Questionnaire (GHQ-12; Goldberg and Williams, 1988), which is “Have you been feeling reasonably happy, all things considered?” As happiness is often treated as a unidimensional construct, single-item measures of happiness are very common in subjective well-being research and the validity of such measures is recognized across a large body of literature (Abdel-Khalek, 2006; for a review, see Nor-Azzatunnisak et al., 2017).

#### Lifestyle

Lifestyle was assessed in two ways. First, we measured *changes* in employees’ current lifestyle compared with before the lockdown period. Following our four-component model of lifestyle, we asked respondents the following questions: “Do you sleep well?,” “Do you eat healthily?,” “Do you exercise regularly?,” and “Do you take the time to relax?” Answers were recorded on a 5-point Likert scale ranging from 1 = *much less than usual* to 5 = *much more than usual* (for a similar approach, see Ingram et al., 2020 and Stanton et al., 2020), in line with how the other variables in the model were measured. We created an index for overall lifestyle change by computing the mean of the four items. Lower scores reflect a negative change in lifestyle, while higher scores reflect an improvement in lifestyle (characterized by multiple health behaviors).

Second, we assessed employees’ lifestyle *during* the lockdown period, focusing on those lifestyle behaviors for which guidelines are available (see Kanji et al., 2018). We asked respondents to respond to the questions thinking about most of their days during the corona crisis. For sleep, we asked about hours of sleep, subjective quality of sleep, how often they had been less than 30 min awake during the night, and how often they had felt rested in the morning. To meet the recommended guidelines for healthy sleep, one needs to sleep 7 to 10 h, evaluate one’s sleep quality as good, most of the time or always be less than 30 min awake during the night, and most of the time or always feel rested in the morning (Watson et al., 2015; Ohayon et al., 2017). Only 33 respondents met these criteria for healthy sleep. Therefore, we decided to focus on the first two indicators, namely quantity and subjective quality of sleep, for further analyses. For exercise, we asked respondents about the intensity and amount of their physical activities during the corona crisis. Guidelines prescribe at least 20 min of intensive physical activity (i.e., sports) at least three times a week as well as at least 30 min of moderate intensive physical activity at least five times a week (Kanji et al., 2018). For food, we asked about intake of vegetables and fruit. To meet the recommended guidelines for healthy nutrition, one needs to eat at least 250 g of vegetables and at least two pieces (or 200 g) of fruit (Brink et al., 2016; Kanji et al., 2018). Lifestyle behaviors were dummy coded depending on whether respondents met the criteria for healthy behavior (i.e., 0 = lifestyle risk factor, 1 = meets criteria). We created an index for overall lifestyle during lockdown by computing the sum of the three dummy variables. A lower score reflects an accumulation of lifestyle-related risk factors, while a higher score reflects a healthier lifestyle as it indicates that one meets the recommended guidelines.

#### Control Variables

The survey assessed various demographic variables. In the analyses reported below, we controlled for respondents’ age, gender, relationship status, children at home, care duties, employment status (self-employed or wage worker), organizational tenure, and education. However, following Spector and Brannick (2011), we have performed analyses with and without control variables. The results reported herein are robust to the inclusion of control variables.

### Analytic Approach

Our goal is to test and compare models and illuminate relationships between lifestyle behaviors and the other variables. To test our hypotheses, we used Hayes’ (2018) PROCESS macro (version 3) in SPSS version 26. A stepwise approach was used in which we first tested a mediation model that is not confounded by lifestyle and after that two models in line with the dual role of lifestyle behaviors that we propose herein. Model 4 was used to test the simple mediation model in which blurring of work-life boundaries is negatively related to happiness via emotional exhaustion. We then built on this basic mediation model by adding lifestyle to the model, in two different ways. First, we used model 58 in PROCESS for testing a moderated mediation model with lifestyle as moderator. Importantly, we used lifestyle during the lockdown period as a moderator for the relationships in our model. In the analyses, we examined the role of both overall lifestyle and each of the lifestyle behaviors in influencing employees’ responses to changes in blurring of work-life boundaries. All variables used in the construction of products of predictor variables were mean-centered prior to model estimation. Second, we used model 6 in PROCESS for testing a serial multiple mediator model with lifestyle as second mediator (M_2_). Importantly, we used lifestyle changes as a mediator in this model. Again, we tested for the mediating role of both overall lifestyle and the specific lifestyle behaviors. Bootstrapping with 10.000 samples was used in all analyses and control variables were entered as covariates. We report the unstandardized regression coefficients.

## Results

Before testing the hypotheses, we take a closer look at our study variables to better understand changes associated with the COVID-19 lockdown. Descriptive statistics and correlations are presented in Table 1. In our sample, the COVID-19 lockdown period was associated with higher blurring of work-life boundaries (*M* = 3.50), with 63.5% of respondents reporting that blurring of boundaries is (much) more than usual. We also asked how frequently participants worked from home during this period (*rarely or never*, *sometimes*, *often or always*) and this between-subjects factor was significantly related to changes in blurred boundaries (*F* = 33.7, *p* < 0.001), with those working from home more often experiencing increases in blurring of work-life boundaries. Since the start of the lockdown, 73.6% of respondents had worked from home often or always, compared with 11.1% before the lockdown. While little evidence was found for a negative change in overall lifestyle (*M* = 2.96, with a sum of 1.1 lifestyle behaviors that deteriorated), 26.4% of participants slept worse than usual (*M* = 2.87), 39.5% exercised less than usual (*M* = 2.89), and 29.0% took less time than usual to relax (*M* = 2.97). A positive change in healthy nutrition was observed for 23.1% of participants (*M* = 3.10). These percentages beg the question why the lifestyle behaviors of some have improved while they have deteriorated for others as well as what are the consequences of such changes. As can be seen from Table 1, lifestyle during the COVID-19 lockdown was associated with favorable changes in the other study variables, which preliminarily suggests that a healthy lifestyle enhances employees’ resilience to adverse circumstances.

**TABLE 1:** Descriptive statistics and correlations between study variables.

**Study variables**	***Mean***	***SD***	**1**	**2**	**3**	**4**	**5**
1. Blurred boundaries	3.50	0.64	1				
2. Emotional exhaustion	3.03	0.75	0.30^∗∗^	1			
3. Happiness	3.11	0.65	−0.16^∗∗^	−0.43^∗∗^	1		
4. Lifestyle behaviors^a^	2.96	0.59	−0.22^∗∗^	−0.36^∗∗^	0.30^∗∗^	1	
5. Healthy lifestyle index^b^	1.17	0.95	−0.17^∗∗^	−0.24^∗∗^	0.15^∗∗^	0.44^∗∗^	1

**N*s = 728–760, pairwise. All variables except lifestyle during lockdown refer to changes since the start of the COVID-19 lockdown (March 15, 2020). A mean value of 3 refers to *same as usual*, whereas a value below 3 refers to a decrease and a value above 3 refers to an increase in the respective variable. ^*a*^Changes in sleep, nutrition, physical activity, and relaxation were combined into a lifestyle score. ^*b*^Lifestyle during lockdown is a sum index of the dichotomized variables for sleep, nutrition, and physical activity. The dichotomized variables are coded as 0 = does not meet criteria for healthy lifestyle behavior, 1 = meets criteria for healthy lifestyle behavior. ^∗∗^*p* < 0.01.*

Table 2 provides further information by comparing the key study variables across groups per gender, household composition, and other demographic characteristics. It can be seen that blurring of boundaries was negatively associated with participants’ age (*p* < 0.001) and age of the youngest child (*p* = 0.002). Moreover, an independent samples *t*-test revealed that those with no children at home experienced less deteriorations in blurred boundaries (*t* = −3.44, *p* = 0.001) and lifestyle behaviors (*t* = 2.28, *p* = 0.02) compared with those with one or more children at home. ANOVAs indicated that workers on a permanent contract were worse off in terms of blurred boundaries than the self-employed (*p* = 0.004) and higher level of education was associated with experiences of increased blurring of boundaries (*F* = 18.17, *p* < 0.001) and decreased happiness (*F* = 3.12, *p* = 0.03).

**TABLE 2:** Means of study variables by subgroups of the sample.

**Demographic variable**	**Subgroups**	**Blurred boundaries**	**Emotional exhaustion**	**Happiness**	**Lifestyle behaviors**	**Healthy lifestyle index**
Age		–0.13^∗∗^	0.02	−0.08^∗^	–0.04	0.01
Age of youngest child		–0.17^∗∗^	0.07	–0.08	0.10	0.05
Organizational tenure		–0.05	–0.04	–0.01	–0.01	0.01
Gender	Men	3.53^a^	2.99^a^	3.09^a^	2.98^a^	1.17^a^
	Women	3.49^a^	3.06^a^	3.13^a^	2.94^a^	1.17^a^
Relationship status	Single	3.57^a^	3.13^a^	3.04^a^	2.91^a^	1.05^a^
	In a relationship	3.49^a^	3.01^a^	3.13^a^	2.96^a^	1.19^a^
Household composition	No children at home	3.44^a^	3.01^a^	3.14^a^	3.00^a^	1.18^a^
	At least one child at home	3.59^b^	3.05^a^	3.09^a^	2.90^b^	1.16^a^
Care duties	No	3.52^a^	3.02^a^	3.13^a^	2.96^a^	1.17^a^
	Yes	3.44^a^	3.09^a^	3.01^a^	2.91^a^	1.19^a^
Employment status	Temporary contract	3.46^ab^	3.07^a^	3.13^a^	2.93^a^	1.12^a^
	Permanent contract	3.56^a^	3.04^a^	3.11^a^	2.95^a^	1.14^a^
	Self-employed	3.34^b^	2.94^a^	3.10^a^	3.01^a^	1.30^a^
Education	High school diploma/post-secondary vocational training	3.17^a^	2.92^a^	3.26^a^	2.98^a^	0.98^a^
	Higher vocational training/university bachelor’s degree	3.47^b^	3.02^a^	3.14^ab^	2.96^a^	1.16^a^
	Master’s degree	3.57^b^	3.05^a^	3.08^ab^	2.95^a^	1.21^a^
	Doctoral degree	3.79^c^	3.19^a^	3.00^b^	2.96^a^	1.26^a^

*For interval variables, correlations with study variables are reported. For nominal and ordinal variables, means are reported and compared. Means that do not share superscript letters differ by *p* < 0.05 in pairwise comparisons using Tukey’s HSD. ^∗^p < 0.05. ^∗∗^*p* < 0.01.*

Table 3 shows that changes in the four lifestyle behaviors are interrelated (all *p*s < 0.001). Lifestyle behaviors during the COVID-19 lockdown are also significantly related; the positive correlations between sleep and physical activity [χ^2^(1) = 6.5, *p* = 0.01, Φ_Cramer_ = 0.10], sleep and nutrition [χ^2^(1) = 6.0, *p* = 0.02, Φ_Cramer_ = 0.09], and physical activity and nutrition [χ^2^(1) = 27.8, *p* < 0.001, Φ_Cramer_ = 0.20] suggest that individuals are more likely to meet the criteria for one lifestyle behavior if they meet the criteria for another.

**TABLE 3:** Descriptive statistics and correlations between lifestyle behaviors.

**Lifestyle behaviors**	***Mean***	***SD***	**1**	**2**	**3**	**4**	**5**	**6**	**7**
**Changes**									
1. Sleep	2.87	0.73	1						
2. Nutrition	3.10	0.73	0.23^∗∗^	1					
3. Physical activity	2.89	1.04	0.18^∗∗^	0.44^∗∗^	1				
4. Relaxation	2.97	0.87	0.33^∗∗^	0.29^∗∗^	0.40^∗∗^	1			
**During COVID-19**									
5. Sleep	0.36	0.48	0.37^∗∗^	0.09^∗^	0.08^∗^	0.18^∗∗^	1		
6. Nutrition	0.41	0.49	0.08^∗^	0.15^∗∗^	0.17^∗∗^	0.06	0.09^∗^	1	
7. Physical activity	0.42	0.49	0.15^∗∗^	0.24^∗∗^	0.52^∗∗^	0.29^∗∗^	0.10^∗^	0.20^∗∗^	1

**N*s = 706–749, pairwise. Lifestyle changes refer to changes since the start of the COVID-19 lockdown (March 15, 2020). Scores below 3 reflect a negative change in the respective lifestyle behavior, while scores above 3 reflect an improvement in the lifestyle behavior. Lifestyle behaviors during COVID-19 are dichotomized variables coded as 0 = does not meet criteria for healthy lifestyle behavior, 1 = meets criteria for healthy lifestyle behavior. ^∗^*p* < 0.05, ^∗∗^*p* < 0.01.*

Results from the basic mediation analysis (PROCESS model 4) showed that blurring of work-life boundaries was positively associated with emotional exhaustion (*B* = 0.36, *p* < 0.001), which in turn was negatively related to happiness (*B* = −0.34, *p* < 0.001). The negative indirect effect of blurred boundaries on happiness through emotional exhaustion was also significant (*ab* = −0.12, 95% CI [−0.17, −0.08]), lending support to our mediation hypothesis (H1). The direct effect of blurred boundaries on happiness was not significant (*B* = −0.04, *p* = 0.26).

**FIGURE 2:**
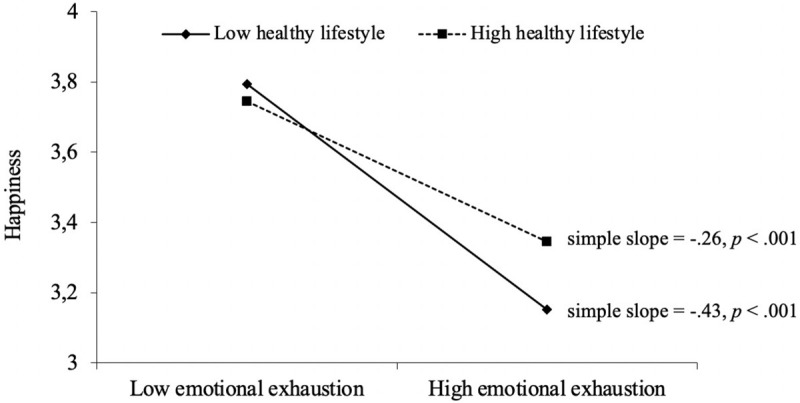
Moderating effect of healthy lifestyle index on the relationship between emotional exhaustion and happiness. Simple slopes are presented for conditional values of the moderator at ±1 SD.

The moderated mediation analysis using PROCESS model 58 revealed that lifestyle was a significant moderator of the association between emotional exhaustion and happiness (*B* = 0.09, *p* = 0.01). This interaction is visually depicted in Figure 2. For the effect of emotional exhaustion on happiness, tests of simple slopes showed that the slopes for both lower (–1 *SD*) and higher (+1 *SD*) levels of a healthy lifestyle were statistically significant. In fact, all obtained values for lifestyle fall within the region of significance of the simple slopes, which defines the specific values of the moderator at which the slope is statistically significant. Thus, a healthy lifestyle cannot entirely prevent emotional exhaustion from decreasing happiness. However, lifestyle does mitigate the detrimental effect of emotional exhaustion on happiness, which is substantiated by results regarding conditional indirect effects; that is, the indirect effect of blurred boundaries on happiness via emotional exhaustion was less negative at higher levels (+1 *SD*) of a healthy overall lifestyle (*ab* = −0.08, 95% CI [−0.13, −0.03]) than at lower levels (–1 *SD*) of a healthy overall lifestyle (*ab* = −0.15, 95% CI [−0.22, −0.08]). Results regarding the moderating role of overall lifestyle are presented in Table 4. The main predictors and control variables together explain 21% of the variance in happiness.

**TABLE 4:** Results of moderated mediation analysis (model 58).

	**Emotional exhaustion (M)**		**Happiness (Y)**	
**Independent variables**	***B***	**SE**	***B***	**SE**
Blurring of work-life boundaries (X)	0.32^∗∗∗^	0.05	–0.03	0.04
Emotional exhaustion (M)			–0.35^∗∗∗^	0.03
Healthy lifestyle (W)	–0.15^∗∗∗^	0.03	0.04	0.02
X × W	–0.03	0.04		
M × W			0.09^∗∗^	0.03
Constant	–0.40	0.21	3.51^∗∗∗^	0.17
Age	0.01	0.003	−0.01^∗^	0.002
Gender^a^	0.09	0.06	0.03	0.05
Relationship status^b^	–0.12	0.07	0.03	0.06
Children at home^c^	0.03	0.06	–0.03	0.05
Care duties^c^	0.04	0.08	–0.03	0.07
Employment status^d^	0.0003	0.08	–0.02	0.07
Tenure (in years)	–0.02	0.02	0.02	0.02
Education	0.04	0.03	–0.07^∗∗^	0.03
	*R*^2^ = 0.14		*R*^2^ = 0.21	
	*F*(11,673) = 10.23, *p* < 0.001		*F*(12,672) = 15.19, *p* < 0.001	

**N* = 685. *B* = unstandardized coefficient. Blurring of work-life boundaries (X), emotional exhaustion (M), and happiness (Y) are variables that refer to changes since the start of the COVID-19 lockdown (March 15, 2020). Healthy lifestyle *during* lockdown is the moderator (W). ^*a*^Gender is coded as 0 = male, 1 = female. ^*b*^Relationship status is coded as 0 = single, 1 = married or in a relationship. ^*c*^Children at home and care duties are coded as 0 = no, 1 = yes. ^*d*^Employment status is coded as 0 = wage worker, 1 = self-employed. ^∗^*p* < 0.05, ^∗∗^*p* < 0.01, ^∗∗∗^*p* < 0.001.*

**FIGURE 3:**
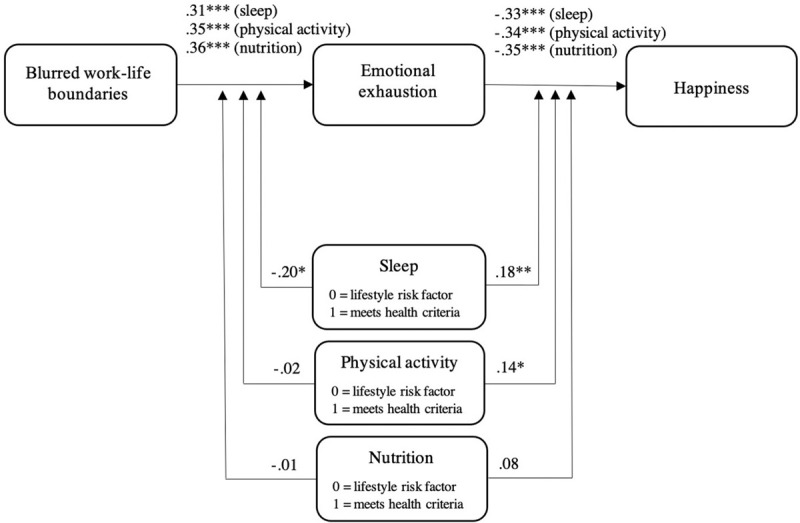
Results of moderated mediation analyses for the various lifestyle behaviors using PROCESS model 58. ^∗^*p* < 0.05, ^∗∗^*p* < 0.01, ^∗∗∗^*p* < 0.001.

Figure 3 presents the results of moderated mediation analyses when zooming in on the specific lifestyle behaviors. We found that sleep was both a significant first-stage moderator for the association between blurred boundaries and emotional exhaustion (*B* = −0.20, *p* = 0.03) and a significant second-stage moderator for the association between emotional exhaustion and happiness (*B* = 0.18, *p* = 0.01). The percentile bootstrap confidence interval for the index of moderated mediation (*B* = 0.11) did not include zero, 95% CI [0.04, 0.18]), indicating that the negative indirect effect of blurred boundaries on happiness was significantly less strong (but still significant) for those who met the criteria for healthy sleep (*ab* = −0.04, 95% CI [−0.08, −0.01]) compared with those who did not meet the criteria (*ab* = −0.15, 95% CI [−0.21, −0.09]). For physical activity, results showed a significant interaction with emotional exhaustion in predicting happiness (*B* = 0.14, *p* = 0.03). However, the confidence interval for the index of moderated mediation (*B* = 0.05) included zero, 95% CI [−0.03, 0.14]), implying that the indirect effect of blurred boundaries on happiness through emotional exhaustion was not conditional on meeting the criteria for healthy physical activity. Finally, results indicated that nutrition did not moderate the association between blurred boundaries and emotional exhaustion (*B* = −0.01, *p* = 0.95), nor the association between emotional exhaustion and happiness (*B* = 0.08, *p* = 0.19). Jointly, though, the results provide evidence for Hypothesis 2 stating that healthier lifestyle behaviors buffer the detrimental indirect effect of blurred boundaries on happiness via emotional exhaustion.

We next tested our serial mediation hypothesis (H3) using PROCESS model 6. This model decomposes the total effect into indirect effects and the remaining direct effect. Three indirect effects were estimated, namely (1) blurred boundaries—emotional exhaustion—happiness, (2) blurred boundaries—lifestyle—happiness, and (3) blurred boundaries—emotional exhaustion—lifestyle—happiness. The results presented in Table 5 indicated that blurred boundaries (*B* = −0.11, *p* = 0.002) and emotional exhaustion (*B* = −0.24, *p* < 0.001) were negatively related to a healthy overall lifestyle, which is turn was positively related to happiness (*B* = 0.17, *p* < 0.001). The effect of blurred boundaries on happiness was significantly mediated in serial by emotional exhaustion and lifestyle, 95% CI [−0.03, −0.01]. The indirect effect of blurred boundaries on happiness through lifestyle but without emotional exhaustion was also significant, 95% CI [−0.04, −0.004], in addition to the mediation path from blurred boundaries through emotional exhaustion to happiness, 95% CI [−0.15, −0.07]. The remaining direct effect of blurred boundaries on happiness was not significant (*B* = −0.02, *p* = 0.52). The model explains 22% of the variance in happiness.

**TABLE 5:** Results from serial mediation analysis (model 6).

	**Emotional exhaustion (M1)**		**Healthy lifestyle (M2)**		**Happiness (Y)**	
**Independent variables**	***B***	**SE**	***B***	**SE**	***B***	**SE**
Blurring of work-life boundaries (X)	0.36^∗∗∗^	0.05	–0.11^∗∗^	0.04	–0.02	0.04
Emotional exhaustion (M1)			–0.24^∗∗∗^	0.03	–0.30^∗∗∗^	0.03
Healthy lifestyle (M2)					0.17^∗∗∗^	0.04
Constant	1.42^∗∗∗^	0.26	4.06^∗∗∗^	0.20	4.00^∗∗∗^	0.27
Age	0.01^∗^	0.003	–0.002	0.002	−0.01^∗^	0.002
Gender^a^	0.10	0.06	–0.01	0.04	0.04	0.05
Relationship status^b^	–0.13	0.08	0.05	0.06	0.04	0.06
Children at home^c^	0.03	0.06	–0.08	0.05	–0.01	0.05
Care duties^c^	0.03	0.08	–0.01	0.07	–0.04	0.07
Employment status^d^	–0.03	0.08	0.003	0.06	–0.01	0.07
Tenure (in years)	–0.02	0.02	0.001	0.02	0.01	0.02
Education	0.03	0.03	0.02	0.03	–0.08^∗∗^	0.03
	*R*^2^ = 0.11		*R*^2^ = 0.14		*R*^2^ = 0.22	
	*F*(9,686) = 9.03, *p* < 0.001		*F*(10,685) = 11.04, *p* < 0.001		*F*(11,684) = 17.98, *p* < 0.001	

**N* = 696. *B* = unstandardized coefficient. Except for the control variables, all variables refer to changes since the start of the COVID-19 lockdown (March 15, 2020). ^*a*^Gender is coded as 0 = male, 1 = female. ^*b*^Relationship status is coded as 0 = single, 1 = married or in a relationship. ^*c*^Children at home and care duties are coded as 0 = no, 1 = yes. ^*d*^Employment status is coded as 0 = wage worker, 1 = self-employed. ^∗^*p* < 0.05, ^∗∗^*p* < 0.01, ^∗∗∗^*p* < 0.001.*

**FIGURE 4:**
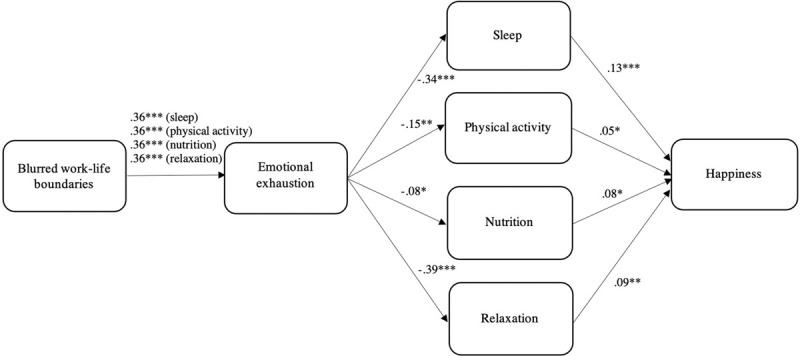
Results of serial mediation analyses for the various lifestyle behaviors using PROCESS model 6. ^∗^*p* < 0.05, ^∗∗^*p* < 0.01, ^∗∗∗^*p* < 0.001.

Regarding the specific lifestyle behaviors, we found that the overall serial mediation involving sleep quality was significant, 95% CI [−0.03, −0.01]. With emotional exhaustion in the model, blurring of work-life boundaries was not associated with sleep quality (*B* = −0.07, *p* = 0.11) and therefore the indirect effect of blurred boundaries on happiness through sleep quality (not involving emotional exhaustion) was not significant, 95% CI [−0.03, 0.004]. The mediation path from blurred boundaries through emotional exhaustion to happiness was significant, 95% CI [−0.15, −0.07], and the remaining direct effect of blurred boundaries on happiness was not significant (*B* = −0.04, *p* = 0.34). Physical activity was also a significant mediator in the total serial mediation path, 95% CI [−0.01, −0.0001]. Blurring of boundaries was not directly associated with physical activity (*B* = −0.04, *p* = 0.32) and its indirect effect on happiness via physical activity was therefore also not significant, 95% CI [−0.02, 0.003]. The mediation path from blurred boundaries through emotional exhaustion to happiness was significant, 95% CI [−0.16, −0.08], and the remaining direct effect of blurred boundaries on happiness was not significant (*B* = −0.04, *p* = 0.32). For nutrition, the results showed that both blurred boundaries (*B* = −0.14, *p* = 0.01) and emotional exhaustion (*B* = −0.08, *p* = 0.048) were negatively related to healthy eating. However, the percentile bootstrap confidence interval for the total serial mediation path (only just) included zero and was therefore not significant, 95% CI [−0.01, 0.0003]. The indirect effect of blurred boundaries on happiness through nutrition (not involving emotional exhaustion) was significant, 95% CI [−0.02, −0.0001], as well as the mediation path from blurred boundaries through emotional exhaustion to happiness, 95% CI [−0.16, −0.08]. The remaining direct effect of blurred boundaries on happiness was not significant (*B* = −0.03, *p* = 0.38). Finally, all indirect effects involving relaxation were significant. Results indicated that the effect of blurred boundaries on happiness was significantly mediated in serial by emotional exhaustion and relaxation, 95% CI [−0.02, −0.004]. The indirect effect of blurred boundaries on happiness through relaxation but without emotional exhaustion was also significant, 95% CI [−0.03, −0.002], as well as the mediation path from blurred boundaries through emotional exhaustion to happiness, 95% CI [−0.15, −0.07]. As in the other models, the remaining direct effect of blurred boundaries on happiness was not significant (*B* = −0.03, *p* = 0.44). Jointly, the results of our serial mediation analyses provide strong support for Hypothesis 3. Figure 4 offers a parsimonious presentation of the serial mediation results involving the various lifestyle behaviors.

## Discussion

The COVID-19 pandemic has compelled many employees worldwide to work from home. Experiences gained during lockdown are likely to have long-lasting consequences. For instance, 57% of the participants in our sample reported they would like to work from home more often in the future. Moreover, 46% of participants expected that supervisors and co-workers would be more supportive of them working from home compared with before the lockdown. Given that the blurring of work-life boundaries gets intensified for home workers, as we have found in our study and other scholars have argued as well (e.g., Bouziri et al., 2020), we set out to examine the effect of blurred boundaries on subjective well-being (i.e., happiness). In a sample of employees in the Netherlands, we observed that increases in blurred work-life boundaries predicted negative changes in happiness through enhanced emotional exhaustion.

Yet the main goal of this study was to illuminate the role of lifestyle in the process by which blurred work-life boundaries decrease happiness (see Figure 1). We found that a healthy lifestyle acted as a buffer for the detrimental indirect effect of blurred work-life boundaries on happiness. That is, those who exhibited a healthy lifestyle (characterized by multiple health behaviors) during lockdown were less negatively affected by increases in the blurring of work-life boundaries in the form of reduced happiness. Sleep in particular was a protective lifestyle factor; both the effect of blurred boundaries on emotional exhaustion and the effect of emotional exhaustion on happiness were weakened for those whose sleep had been adequate (i.e., meeting health guidelines) during the COVID-19 lockdown. Physical activity was also a protective lifestyle behavior; those who had been physically active during lockdown were less likely to suffer from lower happiness as a consequence of emotional exhaustion. However, physical activity did not buffer the indirect effect of blurred boundaries on happiness through emotional exhaustion.

We can conclude that lifestyle is an important buffer for those who experience blurred work-life boundaries and are emotionally exhausted from work. Somewhat paradoxically, however, we observed that blurred boundaries and emotional exhaustion make a healthy lifestyle more unlikely. Those who experienced heightened levels of blurring of work-life boundaries during the COVID-19 lockdown period reported a deterioration in lifestyle behaviors. Deterioration in lifestyle in turn was related to poor subjective well-being in terms of reduced happiness. This mediation effect holds for all lifestyle behaviors in our model; that is, sleep, physical activity, nutrition, and relaxation were all directly or indirectly (via emotional exhaustion) negatively affected by increases in the blurring of work-life boundaries and they all contributed to happiness. In other words, changes in lifestyle behaviors underpin the detrimental effect of blurred boundaries on happiness.

It appears that, although the need for a healthy lifestyle increases when work-life boundaries become blurred, the most likely response to this blurring seems engaging in unhealthy behaviors. Employees who experience blurring of work-life boundaries are at risk of poor sleep and neglecting relaxation, physical activity, and healthy eating. Given our results on the protective function of a healthy lifestyle, this response can be considered maladaptive and dysfunctional. For individuals who experience blurred work-life boundaries, it is particularly important to have a good night’s rest and (to a lesser degree) move and exercise regularly. When these health-promoting behaviors are not encouraged as part of the work and family climates (Buden et al., 2017), a paradox might occur in which those employees who would benefit the most from a healthy lifestyle are less able to lead a healthy life.

### Contributions

Our study contributes to literature on the work-family interface and to research on role blurring and boundary dynamics more specifically. While a substantial body of research investigates work-family conflict (Allen et al., 2000) and work-life balance (Sirgy and Lee, 2018), only few studies have been conducted on the consequences of the blurring of boundaries between work and private life. In this paper, we have shown that blurred work-life boundaries – as an emotionally exhausting experience – reduce happiness. We believe this is an important finding that adds to the growing body of research on the impact of COVID-19 on work and well-being. Importantly, O’Connor et al. (2020) have identified remote and flexible working arrangements as one of the research priority domains for the COVID-19 pandemic for psychological science. Ultimately, our study results allow for a more nuanced understanding of the consequences of working from home as new lockdown situations have occurred in many countries worldwide and employers and employees plan and prepare for new (possibly hybrid) ways of working in a post-pandemic world.

Given that current research priorities are related to addressing the negative biopsychosocial effects of the COVID-19 pandemic (O’Connor et al., 2020), taking a closer look at lifestyle (i.e., sleeping, eating, exercising, relaxation) and how such health behaviors may counter adverse effects is timely (Balanzá-Martínez et al., 2020). As Figure 1 illustrates, our focus on lifestyle enhances our understanding of why (i.e., mediators) and under which circumstances (i.e., moderators) blurring of work-life boundaries is associated with lower happiness. The current study builds on lifestyle research that investigates the relationship between lifestyle behaviors and work-related factors (e.g., Robroek et al., 2013; Van Scheppingen et al., 2015; Larsson et al., 2019). We are among the first to show that a healthy lifestyle can safeguard employees’ well-being in the face of blurring of work-life boundaries and job strain. This result has important practical implications, and the importance of sleep in particular should be emphasized, based on both the results of the present study and other research that has identified sleep as a crucial lifestyle contributor to health (e.g., Ding et al., 2014). Notoriously, sleep problems in the general population are a worldwide concern (Léger et al., 2008).

### Practical Implications and Recommendations

As the world of work is changing, employees face a mosaic of old and new challenges. The majority of our participants intend to work from home more often in the wake of the pandemic. For employees, greater discretion and fungibility of time use (Borpujari et al., 2020), fewer interruptions and commute struggles (Haddad et al., 2009), and smoother transitions between work and family roles (Ashfort et al., 2000) are among the many benefits of working from home. However, working from home comes with blurring of work-life boundaries, which is among the most commonly identified occupational hazards. Our study identifies a major disadvantage and risk associated with the blurring of work-life boundaries that comes with working from home; we found that lifestyle deteriorates, putting employees’ happiness and subjective well-being in jeopardy.

Jointly, the results presented herein suggest that employees – in particular home workers – may find themselves in a vicious spiral. Not only are those for whom a healthy lifestyle would be most beneficial less able (cf. willing) to sustain a healthy lifestyle, but research also shows that unhappy individuals live a less healthy life, which in turn leads to lower happiness (Trudel-Fitzgerald et al., 2019). To break the vicious spiral, work-life struggles, lifestyle, and happiness can be targeted.

First of all, it seems evident that strategies must be developed to overcome blurring of boundaries and the associated emotional exhaustion. The COVID-19 lockdown has created a rather unique situation in which many employees face a new level of work-life integration that undoubtedly requires novel strategies, especially if childcare or homeschooling has to be provided and if individuals work in the same space, and perhaps even with the same resources, as their family members (Cho, 2020). As offices begin to re-open, employers and employees have to consider ways to prepare for the new normal. Multiple strategies to decrease work-related and work-life struggles have been suggested in the literature, such as alternating between hard work and relaxation (i.e., time shifting), setting clear priorities both professionally and personally to manage boundaries (i.e., goal setting), countering negative thoughts and feelings like guilt about blurring of boundaries (i.e., cognitive reframing), and seeking support (Chittenden and Ritchie, 2011; Blake et al., 2020). Another strategy to decrease blurring of boundaries for people working from home, especially those with high family demands, is adapting working time and schedule (Bouziri et al., 2020). Lopez-Leon et al. (2020) listed twelve recommendations that might be helpful for home workers who experience work-life struggles, including avoiding multitasking.

Some of the recommended strategies for home workers focus on taking good care of oneself and being balanced (Chittenden and Ritchie, 2011; Blake et al., 2020; Lopez-Leon et al., 2020). Indeed, it is critical that a health-promoting lifestyle remains a priority. The results by López-Bueno et al. (2020) suggest that people might gradually adapt to circumstances and improve lifestyle behaviors with longer confinement. Lifestyle changes can consist of being more physically active, reducing sedentary time, eating healthier (e.g., more fruits and vegetables), seeking relaxation, giving and receiving social support, improving sleep routines, quit smoking, and drinking alcohol only in moderation. Family members, co-workers, employers, coaches, and therapists can help with and support such lifestyle changes (Verheijden et al., 2005; Walsh, 2011; Buden et al., 2017; Van Rinsum et al., 2018).

We believe our findings regarding the influence of work on lifestyle points in particular to a role for employers; that is, facilitating a healthy lifestyle and well-being among employees should be considered a shared responsibility between employers and employees. Organizations can encourage a healthy lifestyle by developing a so-called organizational health behavior climate (Sonnentag and Pundt, 2016). Organizations will benefit in terms of better work-related outcomes, as suggested by research demonstrating that workplace health programs focusing on healthy nutrition and physical activity can result in better work-related outcomes, such as higher work engagement, work ability, productivity, and performance (Chen et al., 2015; Grimani et al., 2019; Jindo et al., 2020). Moreover, unhealthy lifestyle behaviors render sickness absence-related costs for employers, with sleep problems having the highest financial impact (Kanerva et al., 2018). When people are working from home, specific health-promoting behaviors that supervisors and co-workers can encourage are creating a morning routine that consists of exercises or going for a walk (Lopez-Leon et al., 2020) followed by a healthy breakfast (Ferrer-Cascales et al., 2018), scheduling breaks and lunch time (Miron and Colosi, 2018), socializing with colleagues (Buden et al., 2017), and maintaining regular work hours (Buden et al., 2017), after which (or amidst) one should have recovery experiences such as described by Sonnentag and Fritz (2015). In sum, employees are strongly advised to adapt their lifestyle to working from home rather than letting working from home adapt their lifestyle.

Finally, happiness is a point of interest when advising people who experience blurring of work-life boundaries. We investigated happiness as a subjective well-being outcome of a healthy lifestyle, but lifestyle and happiness most likely have a bidirectional and reciprocal relationship (Trudel-Fitzgerald et al., 2019). Hence, happiness can be considered a modifiable determinant of lifestyle. Strategies to lead a happier life can consist of expressing gratitude, being optimistic, and engaging in prosocial behaviors (Myers and Diener, 2018).

### Limitations and Future Research Directions

The present study is not without its limitations. First, our sample might not be representative in some respects due to our use of a snowball sampling strategy. For instance, most participants in the current research were highly educated. Online participant recruitment and data collection may also have resulted in a non-random sample of the population. Second, concerns related to cross-sectional designs (i.e., causal claims are not warranted) and the use of self-reported variables (i.e., common-method bias) apply to our study. We recommend future research to test our model using a longitudinal design, which would not only overcome some of the limitations of the current research but also allow for examining reciprocal and spiral effects among our study variables over time. Another fruitful endeavor would be to employ a daily diary design because some of the processes examined in this paper are inherently manifested at the intraindividual level. Examining momentary states of employees allows researchers to identify the day-to-day triggers of (un)healthy lifestyle behaviors and shed light on why those who are in dire need of a healthy lifestyle are less able to sustain health-promoting behaviors. A third limitation, specific to data collection during the COVID-19 pandemic, is that changes occurred since the start of the lockdown period, such as Dutch childcare centers re-opening on May 11 (Government of the Netherlands, 2020). Because such changes could have influenced people’s experiences, it might have made it more difficult for participants to respond retrospectively to our survey questions as they applied to the total lockdown period since March 15. Viewed more generally, recall bias poses a threat to the validity of the study findings because of the retrospective design. Another potential limitation relates to the use of a single-item measure for happiness as a global evaluation of happiness over a certain period of time. Happiness is increasingly recognized as a complex and multifaceted phenomenon (Diener et al., 2009), and we therefore recommend future research to adopt a composite measure for a more full-fledged understanding of how happiness is affected. Finally, we did not measure individuals’ boundary management preferences, even though the lockdown period is likely to pose more challenges for people who prefer work-family segmentation (Cho, 2020). Future research is encouraged to test our hypotheses after the COVID-19 situation passes and extend our model with additional mediators and moderators. Modeling additional variables may help to more precisely predict lifestyle behaviors and happiness, as the explained variances of the models tested in the current study were relatively low.

## Conclusion

Following recent calls by Balanzá-Martínez et al. (2020) and Cho (2020), we have integrated in this paper a focus on blurring of work-life boundaries and lifestyle behaviors in the context of the COVID-19 pandemic. The current research has used data on the first wave of COVID-19 in the Netherlands and found evidence for a dual role of lifestyle in the proposed pathway linking blurred work-life boundaries to lower happiness. We have established that healthy lifestyle behaviors (in particular sleep and physical activity) can buffer employees against the detrimental effects of blurred work-life boundaries and emotional exhaustion on happiness. Yet we also found that greater blurring of work-life boundaries makes it more difficult for employees to sustain a healthy lifestyle (in terms of sleep, nutrition, physical activity, and relaxation). Our results regarding the influence of work-related factors on lifestyle behaviors suggest that, for many employees, it is a matter of not being able (versus willing) to lead a healthy life. The current research has important implications for both employees and employers, as we argue that lifestyle and well-being should be considered a shared responsibility between them.

## Data Availability Statement

The data supporting the conclusions of this study are available via https://doi.org/10.17026/dans-xzj-hzz6.

## Ethics Statement

Ethical review and approval was not required for the study on human participants in accordance with the local legislation and institutional requirements. Written informed consent for participation was not required for this study in accordance with the national legislation and the institutional requirements.

## Author Contributions

HP and JW contributed to the conception and design of the study. HP and JW contributed to data collection. HP organized the database and performed the statistical analyses. HP and JW discussed and interpreted the results. JW carried out the literature search. HP took the lead in writing the original draft of the manuscript with input from JW. All authors contributed to the article and approved the submitted version.

## Conflict of Interest

JW at the time of study was employed by company SMC Rijnland Fysiotherapeuten. The remaining authors declare that the research was conducted in the absence of any commercial or financial relationships that could be construed as a potential conflict of interest.
